# Henry Ernest Hetling

**Published:** 1924-01

**Authors:** 


HENRY ERNEST HETLING, L.R.C.P., L.R.C.S. Edin.

We regret to record the death, at the ripe age of 92, of one of the founders of the Bristol Medico-Chirurgical Society. Henry Ernest was the grandson of William Hetling, who was Surgeon to the Bristol Royal Infirmary from 1807 to 1837, and concerning whom much interesting matter is to be found in Munro Smith's history of that institution.

Many of his old friends have gone from our midst, but among the Bristol practitioners who remember him in his active work his memory is cherished.

We are indebted to Mr. W. H. Harsant for the following note:—

He was the son of W. E. Hetling, and he began life as a clerk in the Great Western Railway Company. He entered the Bristol Medical School as a student in 1864, and took the qualifications of L.R.C.P. and L.R.C.S. of Edinburgh. He first held the post of House Surgeon to the Bristol Hospital for Sick Children, and afterwards, having been appointed Surgeon (1869–71) to the Union Mining Company of Newfoundland, he went to that Colony, where he lived several years and visited many parts of Canada. On his return to England he at first began practice in London, but soon afterwards, in 1874, obtained the appointment of Surgeon to the Bristol Dispensary and came to live in Bristol. He finally retired from practice a few years ago. For many years he was a regular attendant at the meetings of the Medico-Chirurgical Society, and was much valued as a friend by some of the old members.

